# Post-peak dependence analysis in statistical damage constitutive modeling of brittle rocks

**DOI:** 10.1038/s41598-026-49872-7

**Published:** 2026-04-26

**Authors:** Yixiao Shen, Peidong Zhu, Chao Zhang, Hao Zhou, Dongping Zhu, Yongyi Li

**Affiliations:** 1 Department of Civil Engineering, University of Hong Kong, Hong Kong, 999077 China; 2 College of Civil Engineering, Hunan University of Science and Technology, Xiangtan, 411201 China; 3 China Railway No.5 Engineering Group Co.,Ltd., Changsha, 410114 China

**Keywords:** Brittle rocks, Statistical damage constitutive model, Probability distribution, Strength criterion, Relative brittleness index, Post-peak region, Engineering, Materials science, Solid Earth sciences

## Abstract

Accurately simulating the post-peak behavior of brittle rocks remains a challenge for statistical damage constitutive models. This study investigates the sensitivity of post-peak simulation to model components by evaluating combinations of three strength criteria and three micro-element strength distributions against triaxial test data from five rock types. Results indicate that the probability distribution of micro-element strength governs simulation accuracy, while the strength criterion exerts minimal influence. Based on these findings, a relative brittleness index-based method for selecting the optimal distribution is proposed, along with a dual-parameter collaborative correction method. Validation shows that this approach significantly enhances the fit to experimental post-peak curves, eliminates criterion-induced interference in parameter adjustment, and remains effective across multiple distribution modes. The proposed method offers a solution for improving the reliability of damage models in predicting brittle rocks failure.

## Introduction

As a naturally occurring geological material, rock inherently contains numerous randomly distributed micro-defects, which lead to highly complex mechanical responses under external loading^1–3^. Triaxial compression tests demonstrate that brittle rocks generally progress through sequential stages: elastic deformation, plastic yielding and post-peak softening^4,5^. Due to the fact that catastrophic failure in brittle rocks often occurs abruptly without apparent precursors, accurately characterizing the post-peak mechanical behavior is essential for stability analysis and risk assessment in rock engineering.

Recent progress in servo-controlled testing techniques has enabled accurate acquisition of complete stress–strain curves, thereby providing reliable test data for in-depth investigation of rock mechanical behavior. However, constitutive models established based on classical elastoplastic theory remain inadequate in capturing the increasingly complex responses of rocks^6^. In recent years, statistical damage theory framework has been established by integrating strength statistics with continuum damage mechanics, founded on the strain equivalence hypothesis and the effective stress principle. Subsequent developments within this framework have produced a variety of statistical damage constitutive models. Current research primarily focuses on five interrelated aspects: damage thresholds and stress-state effects^7,8^, quantification methods for element strength^9–11^, stochastic distribution patterns of element strength^12–14^, determination methods for model parameters^15–17^ and simulation approaches for the residual strength stage^18,19^. Collectively, these efforts have significantly advanced statistical damage constitutive theory and markedly improved its capability to simulate the complete deformation and failure process of brittle rocks.

Despite progress in constructing and refining the statistical damage model framework, existing models still inadequately characterize the post-peak stress degradation behavior of brittle rocks. Specifically, few studies have systematically evaluated model performance in simulating post-failure mechanical behavior across rocks with different brittleness levels. This limitation is particularly evident in the selection of characterization methods for element strength and stochastic distribution patterns, where brittleness-dependent effects on theoretical curves are routinely neglected^14^. Moreover, the distribution patterns for micro-element strength in current models largely rely on empirical assumptions, introducing considerable subjectivity while lacking unified selection criteria or theoretical justification. Such arbitrary selection lead to significant deviations between post-peak theoretical curves and test data, thereby severely limiting the predictive capability of these models under complex field conditions. To date, no study has comprehensively examined the evolution patterns and influencing factors of post-peak theoretical curves within statistical damage models, nor has it established effective modification methods capable of rapidly adjusting these curves to accurately simulate stress degradation across different rock brittleness grades.

This study adopts three widely used rock strength criteria (the Mohr–Coulomb, Drucker–Prager and Hoek–Brown criterion), coupled with three commonly adopted probabilistic distributions for micro-element strength (the Weibull, Normal and Log-normal distribution) to simulate triaxial compression test data from five different lithological types. Through the approach, this paper rigorously analyzes the post-peak characteristics of brittle rocks damage constitutive models. Subsequently, a dual-parameter coordinated modification method is developed, which substantially improves the accuracy of theoretical models in predicting experimental post-peak curves. This contributes to a more robust theoretical method for stability analysis and risk assessment in rock engineering.

## Damage model of brittle rocks

Damage models form a fundamental component in the development of constitutive models based on damage mechanics. Classical damage frameworks are essentially built upon Lemaitre’s strain equivalence hypothesis^20,21^, which posits that damage originates from internal voids within rocks. These voids and the intact matrix collectively constitute the rock medium, with the damage factor *D* serving to relate the nominal stress $\sigma_{i}$(*i*, *j*, *k* = 1, 2, 3) to the effective stress $\sigma^{\prime}_{i}$(*i*,* j*, *k* = 1, 2, 3) as follows:

1 $$\sigma_{i} = \sigma_{i}^{\prime} (1 - D)$$

Equation (1) indicates that the evolution of damage, reflected by an increase in the damage variable *D*, corresponds to a gradual degradation of bearing capacity. In the theoretical limit of complete damage (*D* = 1), the nominal stress reduces to zero^22^. Although this formulation captures certain macroscopic deformation and fracture phenomena induced by damage, it does not account for the residual strength that is consistently observed in experiments on brittle rocks. To address this discrepancy, Cao et al.^23^ argued that damage should not be solely attributed to the formation of voids. They incorporated a persistent residual strength, denoted as $\sigma^{\prime\prime}_{i}$ (*i*,* j*, *k* = 1, 2, 3), and proposed a modified damage model as follows:

2 $$\sigma_{i} = \sigma_{i}^{\prime } (1 - D) + \sigma_{i}^{\prime \prime } D$$

Equation (2) indicates that even at a state of complete damage (*D* = 1), rocks retain a certain load-bearing capacity, represented by $\sigma^{\prime\prime}_{i}$. However, the model assumes that this residual capacity degrades progressively with continued deformation. Consequently, the $\sigma^{\prime\prime}_{i}$ asymptotically approaches zero, resulting ultimately in a null residual stress at failure. Therefore, this formulation fails to adequately explain the persistence of residual strength observed in post-failure rock or to characterize its subsequent stability behavior. To establish a more physically representative damage model, Zhao et al.^24^ proposed the following fundamental hypotheses:

- Rocks can be represented as a composite material comprising undamaged and damaged components that collectively sustain applied stresses.
- The undamaged component obeys the generalized Hooke’s law, with its stress–strain relationship defined by the elastic modulus *E* and Poisson’s ratio $\nu$s, as follows:
  3 $$\sigma_{i}^{\prime } = E\varepsilon_{i}^{\prime } + \nu (\sigma_{j}^{\prime } + \sigma_{k}^{\prime } )$$
  where $\varepsilon^{\prime}_{i}$(*i*, *j*, *k* = 1, 2, 3) is the microscopic strain of undamaged component. Given the nominal strain of rocks as $\varepsilon_{i}$ (*i*, *j*, *k* = 1, 2, 3) and the microscopic strain of damaged components as $\varepsilon_{i}^{r}$(*i*, *j*, *k* = 1, 2, 3), the strain compatibility principle yields:
  4 $$\varepsilon_{i} = \varepsilon_{i}^{\prime } = \varepsilon_{i}^{r}$$
  c. Damage exclusively occurs along the axial direction, while nominal stress equates to effective stress in the lateral directions:
  5 $$\sigma_{j}^{\prime } = \sigma_{j}$$
  6 $$\sigma_{k}^{\prime } = \sigma_{k}$$
  d. When stress attains the damage threshold, rocks undergo rapid damage evolution, subsequently sustaining a stabilized residual stress $\sigma_{i}^{r}$(*i*, *j*, *k* = 1, 2, 3).

Based on the aforementioned postulates, a damage model incorporating residual strength characteristics is derived through micro-mechanical analysis of vertical equilibrium within representative volume element, as follows:

7 $$\sigma_{i} = \sigma_{i}^{\prime } (1 - D) + \sigma_{i}^{r} D$$

Equation (7) demonstrates that when *D* = 1, $\sigma_{i} = \sigma_{i}^{r}$. Evidently, this damage model captures characteristic deformation features of residual strength, outperforming the two aforementioned damage models in representing engineering reality.

## Statistical damage constitutive model of brittle rocks

By substituting Eqs. (4) ~ (6) into Eq. (3), we obtain:

8 $$\sigma_{i}^{\prime } = E\varepsilon_{i} + \nu (\sigma_{j} + \sigma_{k} )$$

Substituting Eq. (8) into Eq. (7) gives the damage constitutive model for rocks that incorporate residual strength characteristics:

9 $$\sigma_{i} = E\varepsilon_{i} (1 - D) + \lambda D + \nu (\sigma_{j} + \sigma_{k} )$$

in which the notation *λ* is calculated using

10 $$\lambda = \sigma_{i}^{r} - \nu (\sigma_{j} + \sigma_{k} )$$

### Measurement method of micro-element strength

According to statistical damage theory, the damage process in rock materials can be interpreted as follows: during the loading process, the applied stress exceeds the strength of a certain proportion of micro-elements, resulting in their failure, thereby leading to damage evolution at the macroscopic scale^25–27^. Thus, establishing a effective quantification approach for micro-element strength constitutes the basis for characterizing damage evolution process.

Depending on the properties of rocks and the specific engineering context, various strength criteria can be employed to quantify the micro-element strength *F*. In this study, three widely used rock strength criteria are selected, namely the Mohr–Coulomb (M-C), Drucker-Prager (D-P) and Hoek–Brown (H-B) criteria, which are each tailored to distinct geomechanical context for discriminating the strength failure of micro-elements.

- The M-C criterion.

The M-C criterion remains one of the most widely used strength criteria, which is particularly well-suited for brittle materials^28^, expressed as:

11 $$F_{mc} = \sigma_{1}^{\prime } - \alpha {}_{1}\sigma_{3}^{\prime } - k_{1}$$

in which the notations *α*_1_ and *k*_1_ are calculated using, respectively

12 $$\alpha_{1} = \frac{{1 + \sin \phi_{y} }}{{1 - \sin \phi_{y} }}$$

13 $$k_{1} = \frac{{2c_{y} \cos \phi_{y} }}{{1 - \sin \phi_{y} }}$$

where *c*_*y*_ and *ϕ*_*y*_ are the cohesion and the angle of internal friction at yield, respectively.

b. The D-P criterion.

The D-P criterion is a generalized version of the M-C criterion, making it more suitable than the M-C criterion for three-dimensional stress state analyses^18^, formulated as:

14 $$F_{{{\mathrm{dp}}}} = \alpha_{2} I_{1} + \sqrt {J_{2} } - k_{2}$$

in which the notations *I*_1_, *J*_1_, *α*_2_ and *k*_2_ are calculated using, respectively

15 $$I_{1} = \sigma_{1}^{\prime } + \sigma_{2}^{\prime } + \sigma_{3}^{\prime }$$

16 $$J_{1} = \frac{{(\sigma^{\prime}_{1} - \sigma^{\prime}_{2} )^{2} + (\sigma^{\prime}_{2} - \sigma^{\prime}_{3} )^{2} + (\sigma^{\prime}_{1} - \sigma^{\prime}_{3} )^{2} }}{6}$$

17 $$\alpha_{2} = \frac{{\sin \phi_{y} }}{{\sqrt {9 + 3\sin^{2} \phi_{y} } }}$$

18 $$k_{2} = \frac{{\sqrt 3 c_{y} \cos \phi_{y} }}{{\sqrt {9 + 3\sin^{2} \phi_{y} } }}$$

c. The H-B criterion.

The H-B criterion is an empirical and non-linear criterion that is widely employed for characterizing the strength of fractured rock masses^29,30^, expressed as:

19 $$F_{hb} = \sigma_{1}^{\prime } - \sigma_{3}^{\prime } - k_{3}$$

in which the notation *k*_3_ is calculated using

20 $$k_{3} = \sigma_{cy} (m_{cy} \frac{{\sigma_{3}^{\prime } }}{{\sigma_{cy} }} + 1)^{0.5}$$

in which *σ*_cy_ and *m*_cy_ are the uniaxial compressive strength and empirical parameter at yield, respectively.

It should be noted that damage does not occur abruptly upon loading; rather, it initiates only when the applied load reaches a critical level, known as the damage threshold^24^. Therefore, the threshold concept must be accounted for when characterizing micro-element strength. Conventionally, this damage threshold is defined as the minimum effective strength value, denoted *f*_th_, within the statistical distribution of micro-element strengths

21 $$f_{{{\mathrm{th}}}} = \min \{ f_{i} \} ,\;\;\;\;(i = 1,2,...,N)$$

where *f*_*i*_ is the strength of the *i*-th micro-element; *N* is the total number of micro-elements. When the actual stress remains below the *f*_th_, micro-elements remain intact, and the rocks exhibit elastic responses. Once the stress exceeds the threshold, certain micro-elements initiate failure, thereby triggering damage accumulation. Consequently, the theoretical damage thresholds for rocks under the three strength criteria examined in this study are derived as follows:

22 $$f_{{{\mathrm{th}}}} = \left\{ \begin{gathered} k_{1} ,\;\;\;\;F = F_{{{\mathrm{mc}}}} \hfill \\ k_{2} ,\;\;\;F = F_{{{\mathrm{dp}}}} \hfill \\ k_{3} ,\;\;\;F = F_{{{\mathrm{hb}}}} \hfill \\ \end{gathered} \right.$$

Hence, at low stress level (*F* < 0), the rocks remain undamaged (*D* = 0). When the stress reaches the theoretical damage threshold (*F* = 0), the rocks begin to yield but still exhibit no damage (*D* = 0). As the stress progressively increases beyond the threshold (*F* > 0), stochastic damage initiates, leading to the onset of damage accumulation (*D* > 0).

### Damage evolution model

The strength distribution pattern of micro-elements critically determines the damage evolution characteristics of rocks. This study selects three prevalent distribution types: the Weibull distribution^11^, Normal distribution^13^ and Log-normal distribution^14^. Based on statistical damage theory, corresponding damage evolution models are established according to their respective distribution functions.

- The Weibull distribution.

The Weibull distribution (Wd) is commonly employed to characterize the statistical properties of brittle materials strength^11^. Its probability density function is expressed as:

23 $$f_{Wd} (x) = \frac{m}{{F_{0} }}(\frac{x}{{F_{0} }})^{m - 1} \exp [ - (\frac{x}{{F_{0} }})^{m} ]$$

in which *m* and *F*_0_ are the shape parameter and scale parameter, respectively. Under a given loading condition, increasing external load causes progressive failure of micro-elements. The failure probability *P*(X ≤ *F*) of micro-elements can be expressed by the cumulative distribution function:

24 $$P(x \le F) = \int_{0}^{F} {f_{{{\mathrm{Wd}}}} (x)dx}$$

The general expression for the damage evolution model *D*_Wd_ of rocks following the Weibull distribution is obtained by substituting Eq. (23) into Eq. (24):

25 $$D_{{{\mathrm{Wd}}}} = P(F \le 0) = 1 - \exp [ - (\frac{F}{{F_{0} }})^{m} ]$$

Substituting Eqs. (11), (14) and (19) into Eq. (25), respectively, the *D*_Wd_ accounting for the influence of the theoretical damage threshold can be derived under the three strength criteria:

26 $$D_{{{\mathrm{Wd}}}} = \left\{ \begin{gathered} \left\{ \begin{gathered} 1 - \exp [ - (\frac{{F_{{{\mathrm{mc}}}} }}{{F_{0} }})^{m} ],\;\;F_{{{\mathrm{mc}}}} > 0 \hfill \\ 0,\;\;\quad \quad \quad \quad \quad \quad F_{{{\mathrm{mc}}}} \le 0 \hfill \\ \end{gathered} \right.,\quad F = F_{{{\mathrm{mc}}}} \hfill \\ \left\{ \begin{gathered} 1 - \exp [ - (\frac{{F_{{{\mathrm{dp}}}} }}{{F_{0} }})^{m} ],\;\;F_{{{\mathrm{dp}}}} > 0 \hfill \\ 0,\;\;\quad \quad \quad \quad \quad \quad F_{{{\mathrm{dp}}}} \le 0 \hfill \\ \end{gathered} \right.,\quad F = F_{{{\mathrm{dp}}}} \hfill \\ \left\{ \begin{gathered} 1 - \exp [ - (\frac{{F_{{{\mathrm{hb}}}} }}{{F_{0} }})^{m} ],\;\;F_{{{\mathrm{hb}}}} > 0 \hfill \\ 0,\;\;\quad \quad \quad \quad \quad \quad F_{{{\mathrm{hb}}}} \le 0 \hfill \\ \end{gathered} \right.,\quad F = F_{{{\mathrm{hb}}}} \hfill \\ \end{gathered} \right.$$

b. The Normal distribution.

The Normal distribution (Nd) is typically employed to characterize material strength exhibiting a symmetrical pattern. The strength of the majority of micro-elements concentrates near the mean value, whereas extreme values occur with low probability. Its probability density function is expressed as:

27 $$f_{{{\mathrm{Nd}}}} (x) = \frac{1}{{\beta \sqrt {2\pi } }}\exp [ - \frac{{(x - \eta )^{2} }}{{2\beta^{2} }}]$$

where *η* and *β* are the mean strength and standard deviation, respectively. Similarly, the general expression for the damage evolution model *D*_Nd_ of rocks following the Normal distribution can be expressed as:

28 $$D_{{{\mathrm{Nd}}}} = \Phi (\frac{F - \eta }{\beta }) = \frac{1}{{\beta \sqrt {2\pi } }}\int_{0}^{F} {\exp [ - \frac{1}{2}(\frac{x - \eta }{\beta })^{2} ]} dx$$

in which Φ is the cumulative distribution function of the standard normal distribution.

Substituting Eqs. (11), (14) and (19) into Eq. (28), respectively, the *D*_Nd_ for rocks considering the influence of the theoretical damage threshold can be derived under the three strength criteria:

29 $$D_{{{\mathrm{Nd}}}} = \left\{ \begin{gathered} \left\{ \begin{gathered} \Phi (\frac{{F_{{{\mathrm{mc}}}} - \eta }}{\beta }),\;\;F_{{{\mathrm{mc}}}} > 0 \hfill \\ 0,\;\;\quad \quad \quad \quad F_{{{\mathrm{mc}}}} \le 0 \hfill \\ \end{gathered} \right.,\quad F = F_{{{\mathrm{mc}}}} \hfill \\ \left\{ \begin{gathered} \Phi (\frac{{F_{{{\mathrm{dp}}}} - \eta }}{\beta }),\;\;F_{{{\mathrm{dp}}}} > 0 \hfill \\ 0,\;\;\quad \quad \quad \quad F_{{{\mathrm{dp}}}} \le 0 \hfill \\ \end{gathered} \right.,\quad F = F_{{{\mathrm{dp}}}} \hfill \\ \left\{ \begin{gathered} \Phi (\frac{{F_{{{\mathrm{hb}}}} - \eta }}{\beta }),\;\;F_{{{\mathrm{hb}}}} > 0 \hfill \\ 0,\;\;\quad \quad \quad \quad F_{{{\mathrm{hb}}}} \le 0 \hfill \\ \end{gathered} \right.,\quad F = F_{{{\mathrm{hb}}}} \hfill \\ \end{gathered} \right.$$

c. The Log-normal distribution.

Similarly, the general expression for the damage evolution model *D*_Ld_ of rocks following the Log-normal distribution (Ld) can be derived:

30 $$D_{{{\mathrm{Ld}}}} = \Phi [\frac{{\ln (F/S_{0} )}}{\xi }] = \int_{0}^{F} {\frac{1}{{x\xi \sqrt {2\pi } }}\exp [ - \frac{{(\ln x - \ln S_{0} )^{2} }}{{2\xi^{2} }}]} dx$$

in which *ξ* and *S*_0_ are the distribution parameters of the Log-normal distribution.

Substituting Eqs. (11), (14) and (19) into Eq. (30), respectively, the *D*_Ld_ for rocks accounting for the influence of the theoretical damage threshold can be derived under the three strength criteria:

31 $$D_{{{\mathrm{Ld}}}} = \left\{ \begin{gathered} \left\{ \begin{gathered} \Phi (\frac{{\ln (F_{{{\mathrm{mc}}}} /S_{0} )}}{\xi }),\;\;F_{{{\mathrm{mc}}}} > 0 \hfill \\ 0,\;\;\quad \quad \quad \;\quad \quad F_{{{\mathrm{mc}}}} \le 0 \hfill \\ \end{gathered} \right.,\quad F = F_{{{\mathrm{mc}}}} \hfill \\ \left\{ \begin{gathered} \Phi (\frac{{\ln (F_{{{\mathrm{dp}}}} /S_{0} )}}{\xi }),\;\;F_{{{\mathrm{dp}}}} > 0 \hfill \\ 0,\;\;\quad \quad \quad \;\quad \quad F_{{{\mathrm{dp}}}} \le 0 \hfill \\ \end{gathered} \right.,\quad F = F_{{{\mathrm{dp}}}} \hfill \\ \left\{ \begin{gathered} \Phi (\frac{{\ln (F_{{{\mathrm{hb}}}} /S_{0} )}}{\xi }),\;\;F_{{{\mathrm{hb}}}} > 0 \hfill \\ 0,\;\;\quad \quad \quad \;\quad \quad F_{{{\mathrm{hb}}}} \le 0 \hfill \\ \end{gathered} \right.,\quad F = F_{{{\mathrm{hb}}}} \hfill \\ \end{gathered} \right.$$

Substituting Eqs. (26), (29) and (31) into Eq. (9), respectively, the damage constitutive model for rocks considering residual strength characteristics can be obtained under different measurement methods for the strength and probability distribution modes.

## Determination methods of model parameters

The strength distribution of micro-elements exhibits diverse types, including the Weibull, Normal and Log-normal distributions. Thus, determination their distribution parameters becomes a critical challenge in applying statistical damage constitutive models. Current parameter determination methods primarily include the linear fitting method^15^, inverse analysis method^16^ and peak point method^17^. The linear fitting method obtains distribution parameters by fitting discrete test data points. While computationally simple, it lacks physical significance. The inverse analysis method enables high-accuracy parameter determination but requires extensive test datasets. The peak point method exhibits clear physical significance and operational efficiency, rendering it widely applicable. However, this approach necessitates that the statistical damage constitutive model satisfy specific geometric constraint equations:

32 $$\left. {\sigma_{i} (\varepsilon_{i} )} \right|_{{\varepsilon_{i} = \varepsilon_{{{\mathrm{sc}}}} }} = \sigma_{{{\mathrm{sc}}}}$$

33 $$\left. {\frac{{\partial \sigma_{i} }}{{\partial \varepsilon_{i} }}} \right|_{{\sigma_{i} = \sigma_{{{\mathrm{sc}}}} ,\;\varepsilon_{i} = \varepsilon_{{{\mathrm{sc}}}} }} = 0$$

in which *s*_sc_ and *ε*_sc_ are the peak stress and its corresponding strain, respectively. Therefore, Eqs. (32) and (33) not only require the theoretical curve to pass through the peak point of the test curve but also satisfy extremum characteristics. Solving these simultaneous constraint equations yields the distribution parameters. Consequently, the determining equations for the Weibull distribution parameters *m* and *F*_0_ can be expressed as:

34 $$\left\{ \begin{gathered} \frac{m}{{F_{0} }}\exp [ - (\frac{{F_{{{\mathrm{sc}}}} }}{{F_{0} }})^{m} ](\frac{{F_{{{\mathrm{sc}}}} }}{{F_{0} }})^{m - 1} + \frac{{1 - D_{{{\mathrm{sc}}}} }}{{\lambda - E\varepsilon_{{{\mathrm{sc}}}} }} = 0 \hfill \\ 1 - \exp [ - (\frac{{F_{{{\mathrm{sc}}}} }}{{F_{0} }})^{m} ] - D_{{{\mathrm{sc}}}} = 0 \hfill \\ \end{gathered} \right.$$

The determining equations for the Normal distribution parameters *η* and *β* can be expressed as:

35 $$\left\{ \begin{gathered} \frac{1}{\beta }\Phi (\frac{{F_{{{\mathrm{sc}}}} - \eta }}{\beta }) + \frac{{1 - D_{{{\mathrm{sc}}}} }}{{\lambda - E\varepsilon_{{{\mathrm{sc}}}} }} = 0 \hfill \\ \Phi (\frac{{F_{{{\mathrm{sc}}}} - \eta }}{\beta }) - D_{{{\mathrm{sc}}}} = 0 \hfill \\ \end{gathered} \right.$$

The determining equations for the Log-normal distribution parameters *ξ* and *S*_0_

can be expressed as:

36 $$\left\{ \begin{gathered} \frac{a}{{F_{{{\mathrm{sc}}}} }}\Phi (a\ln F_{{{\mathrm{sc}}}} + b) + \frac{{1 - D_{{{\mathrm{sc}}}} }}{{\lambda - E\varepsilon_{{{\mathrm{sc}}}} }} = 0 \hfill \\ \Phi (a\ln F_{{{\mathrm{sc}}}} + b) - D_{{{\mathrm{sc}}}} = 0 \hfill \\ \end{gathered} \right.$$

in which the notations *a* and *b* are calculated using, respectively

37 $$a = 1/\xi$$

38 $$b = - \frac{{\ln S_{0} }}{\xi }$$

in which *F*_sc_ are *D*_sc_ the micro-element strength and damage variable of the rocks at peak stress level, respectively. The explicit expressions are as follows:

For the M-C criterion, *F*_sc_ is expressed as:

39 $$F_{{{\mathrm{sc}}}} = E\varepsilon_{{{\mathrm{sc}}}} + \nu \sigma_{2} + (\nu - \alpha_{1} )\sigma_{3} - k_{1}$$

For the D-P criterion, *F*_sc_ is expressed as:

40 $$F_{{{\mathrm{sc}}}} = \alpha_{2} (\sigma^{\prime}_{1} + \sigma_{2} + \sigma_{3} ) + \sqrt {\frac{{(\sigma^{\prime}_{1} - \sigma_{2} )^{2} + (\sigma_{2} - \sigma_{3} )^{2} + (\sigma^{\prime}_{1} - \sigma_{3} )^{2} }}{6}} - k_{2}$$

in which the notations $\sigma^{\prime}_{1}$ is calculated using

41 $$\sigma^{\prime}_{1} = E\varepsilon_{{{\mathrm{sc}}}} + \nu (\sigma_{2} + \sigma_{3} )$$

For the H-B criterion, *F*_sc_ is expressed as:

42 $$F_{{{\mathrm{sc}}}} = E\varepsilon_{{{\mathrm{sc}}}} + \nu \sigma_{2} + (\nu - 1)\sigma_{3} - k_{3}$$

Substituting the *s*_sc_ and *ε*_sc_ into Eq. (9) yields:

43 $$D_{{{\mathrm{sc}}}} = \frac{{\sigma_{{{\mathrm{sc}}}} - E\varepsilon_{{{\mathrm{sc}}}} - \nu (\sigma_{2} + \sigma_{3} )}}{{\lambda - E\varepsilon_{{{\mathrm{sc}}}} }}$$

The parameter *λ* incorporates the residual stress $\sigma_{i}^{r}$, which can be determined through the corresponding strength criterion. The explicit formulation is as follows:

For the M-C criterion, the residual stress $\sigma_{{{\mathrm{mc}}}}^{r}$ is expressed specifically as:

44 $$\sigma_{{{\mathrm{mc}}}}^{r} = \frac{{(1 + \sin \phi_{r} )\sigma_{3} + 2c_{r} \cos \phi_{r} }}{{1 - \sin \phi_{r} }}$$

in which *c*_r_ and φ_r_ are the cohesion and the angle of internal friction at residual strength, respectively.

For the D-P criterion, the residual stress $\sigma_{{{\mathrm{dp}}}}^{r}$ is expressed as:

45 $$\sigma_{{{\mathrm{dp}}}}^{r} = \frac{{(1 - 2\sqrt 3 \alpha_{2r} )\sigma_{3} + \sqrt 3 k_{2r} }}{{1 + \sqrt 3 \alpha_{2r} }}$$

in which *α*_2r_ and *k*_2r_ are the parameters characterizing rocks at residual strength state.

For the H-B criterion, the residual stress $\sigma_{{{\mathrm{hb}}}}^{r}$ is expressed as:

46 $$\sigma_{{{\mathrm{hb}}}}^{r} = \sigma_{3} + \sigma_{{{\mathrm{cr}}}} (m_{{{\mathrm{cr}}}} \frac{{\sigma_{3} }}{{\sigma_{{{\mathrm{cr}}}} }} + 1)^{0.5}$$

in which σ_cr_ and *m*_cr_ are the uniaxial compressive strength and empirical parameter, respectively.

Consequently, theoretical curves for statistical damage constitutive models that account for residual strength behavior are derived by integrating three strength criteria, three damage evolution models and their corresponding parameter determination methods under various probability distribution modes.

## Post-peak dependence analysis of theoretical models

### Comparative analysis between model curves and test curves

Stress–strain curves for five distinct rock types were obtained using servo-controlled triaxial compression testing system^31–34^. Following standard procedures for processing test data, the deformation and mechanical parameters were summarized in Table 1. By substituting the corresponding data into the proposed model, the proposed model curves can be derived under different strength characterization methods and probability distribution modes.

**Table 1:** The deformation and mechanical parameters for rocks.

Rock	_*E*/GPa_	*v*	φ_y/°_	_*c*y/kPa_	φ_r/°_	_*c*r/kPa_
Coal^31^	3.9	0.19	33.0	7.73	26.0	0.45
Norite^31^	85.0	0.29	60.4	20.99	52.6	5.14
Quartzite^32^	90.0	0.25	30.3	43.97	46.7	5.98
Marble^33^	48.0	0.24	27.7	20.84	36.0	6.62
Sandstone^34^	47.3	0.34	31.9	27.07	30.4	14.96

Rock	*σ*_2r_	*k*_2r_	*σ*_cy_	_*m*cy_	*σ*_cr_	*m*_cr_
Coal^31^	− 0.19	0.36	25.96	7.68	0.01	2146.21
Norite^31^	− 0.42	4.49	147.17	42.07	0.12	7774.77
Quartzite^32^	− 0.37	7.14	156.64	4.16	0.13	6079.69
Marble^33^	− 0.28	9.88	70.88	3.97	0.10	4215.84
Sandstone^34^	− 0.26	12.28	92.30	2.55	0.29	1813.24

**Fig. 1:**
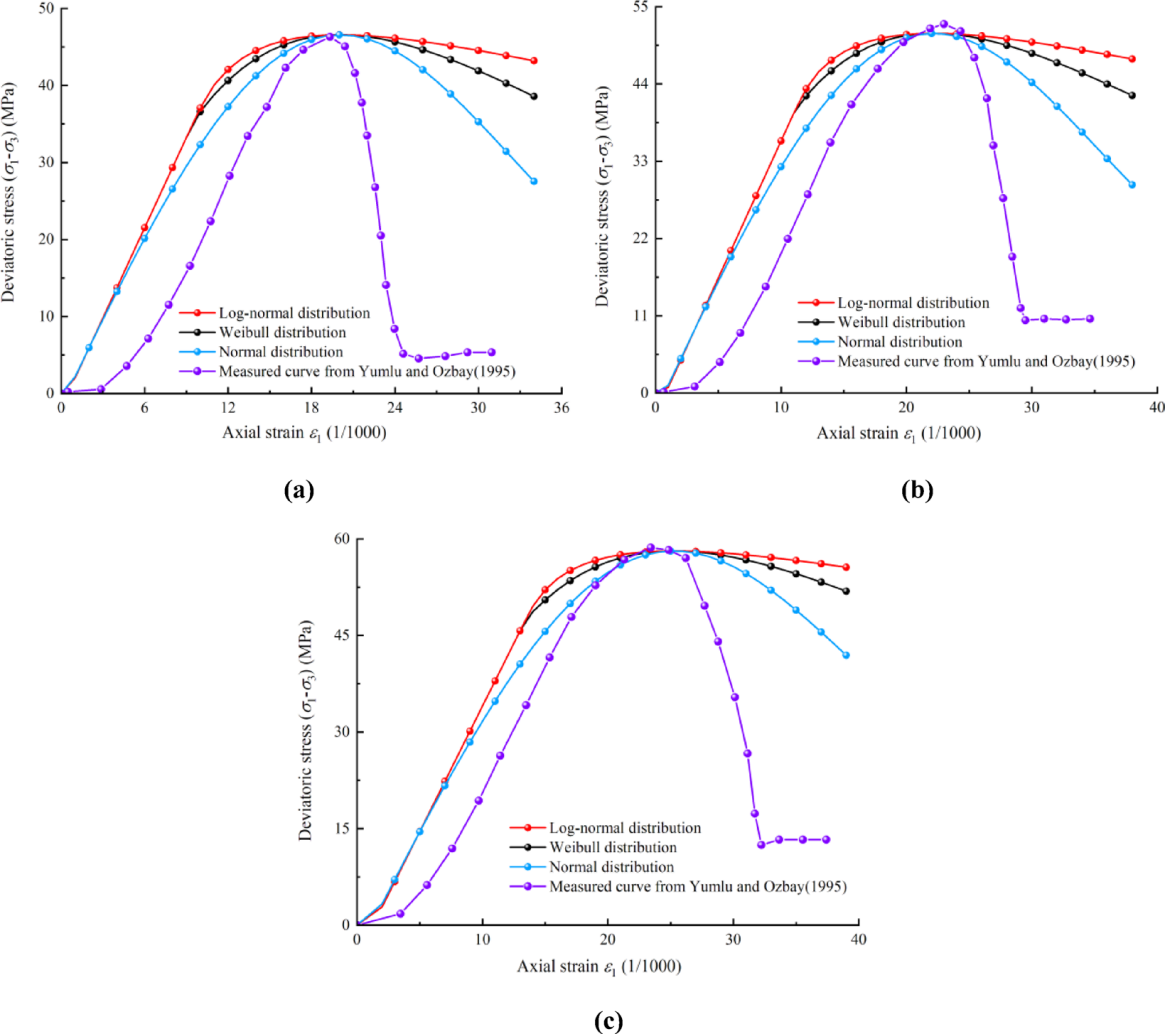
Comparison between measured curves of coal^31^ and probability distribution model curves under different confining pressures. (**a**) σ_3_ = 3.0MPa, (**b**) σ_3_ = 5.0MPa, (**c**) σ_3_ = 8.0MPa.

**Fig. 2:**
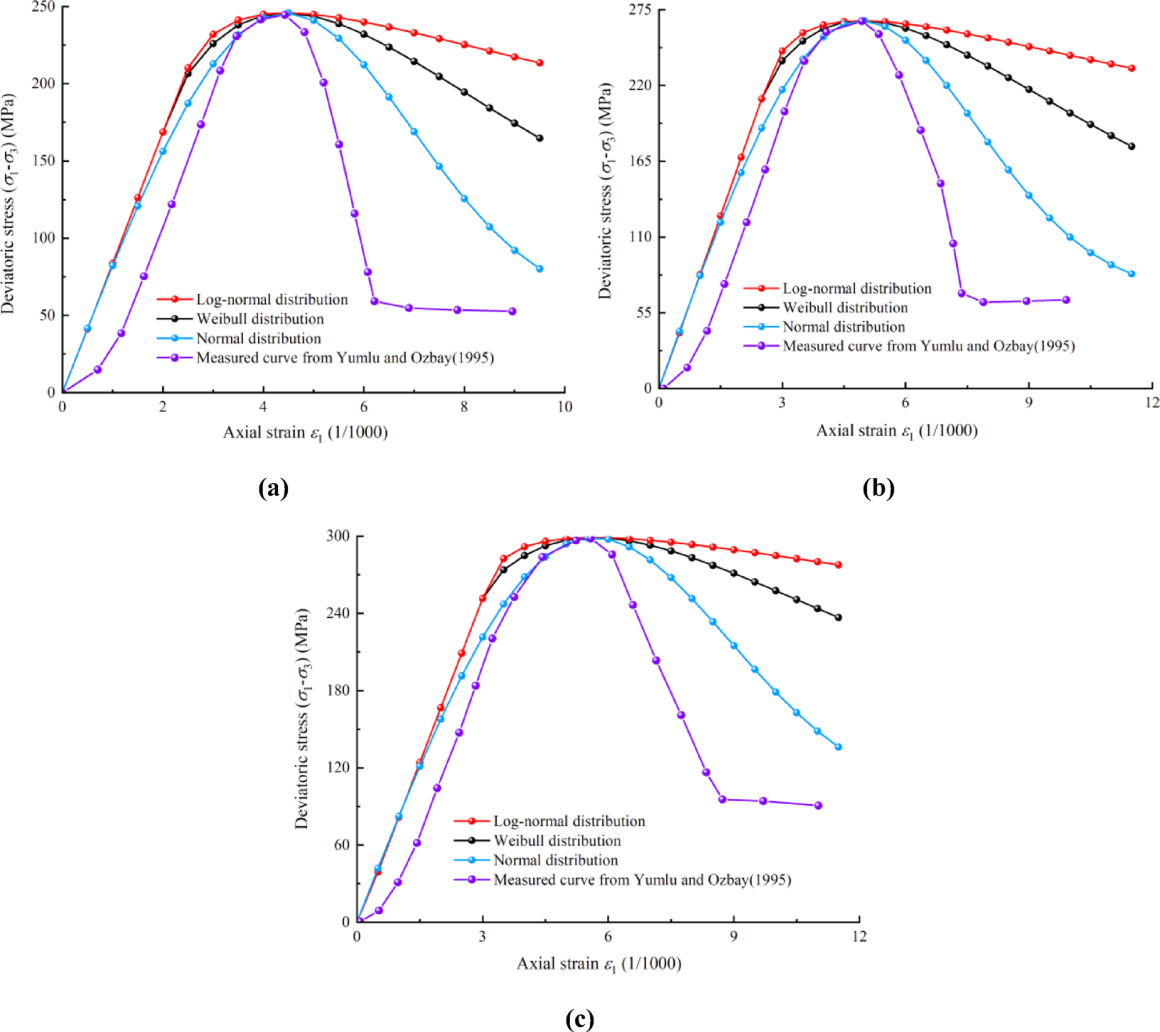
Comparison between measured curves of norite^31^ and probability distribution model curves under different confining pressures. (**a**) σ_3_ = 3.0MPa, (**b**) σ_3_ = 5.0MPa, (**c**) σ_3_ = 8.0MPa.

**Fig. 3:**
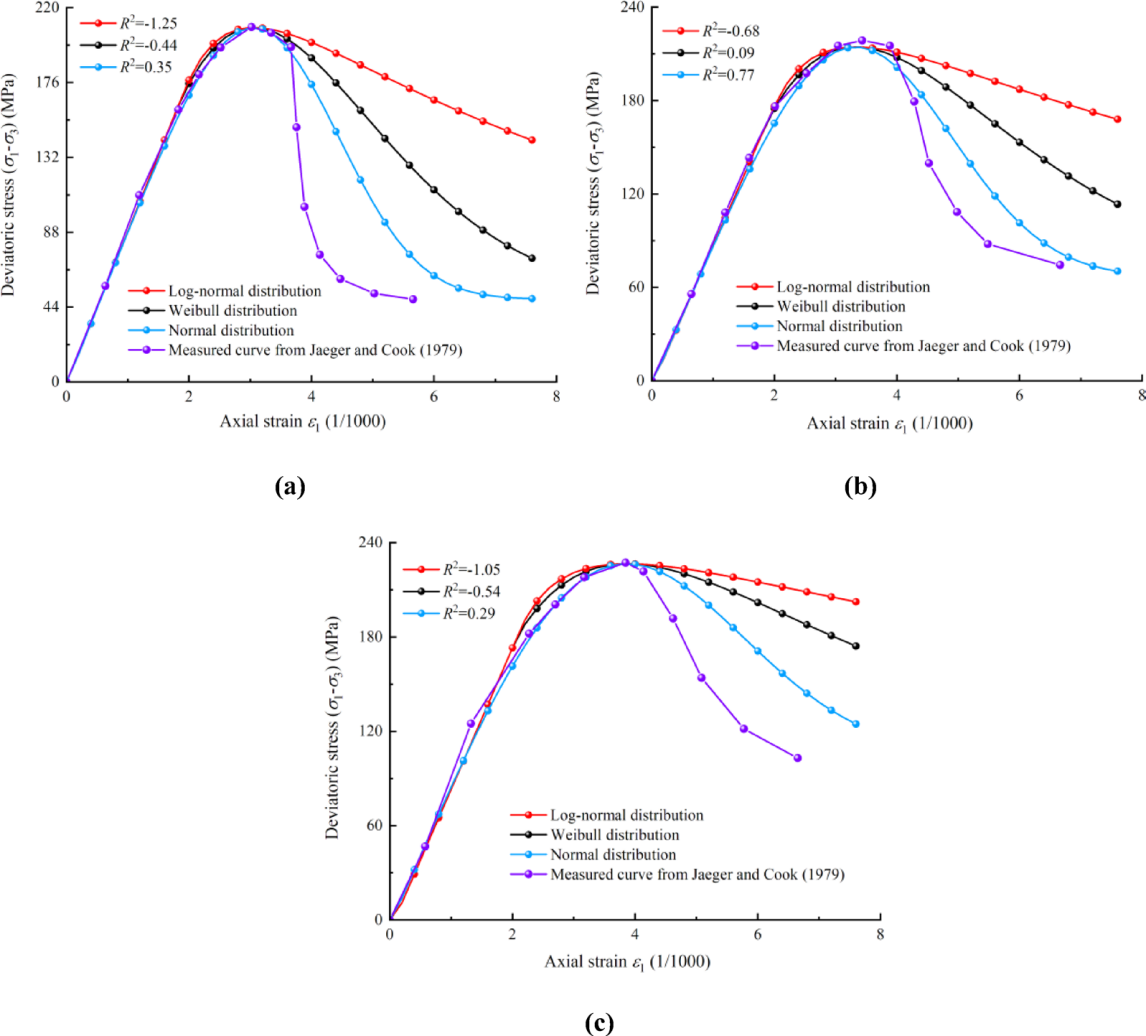
Comparison between measured curves of quartzite^32^ and probability distribution model curves under different confining pressures. (**a**) σ_3_ = 3.45MPa, (**b**) σ_3_ = 6.90MPa, (**c**) σ_3_ = 13.80MPa.

**Fig. 4:**
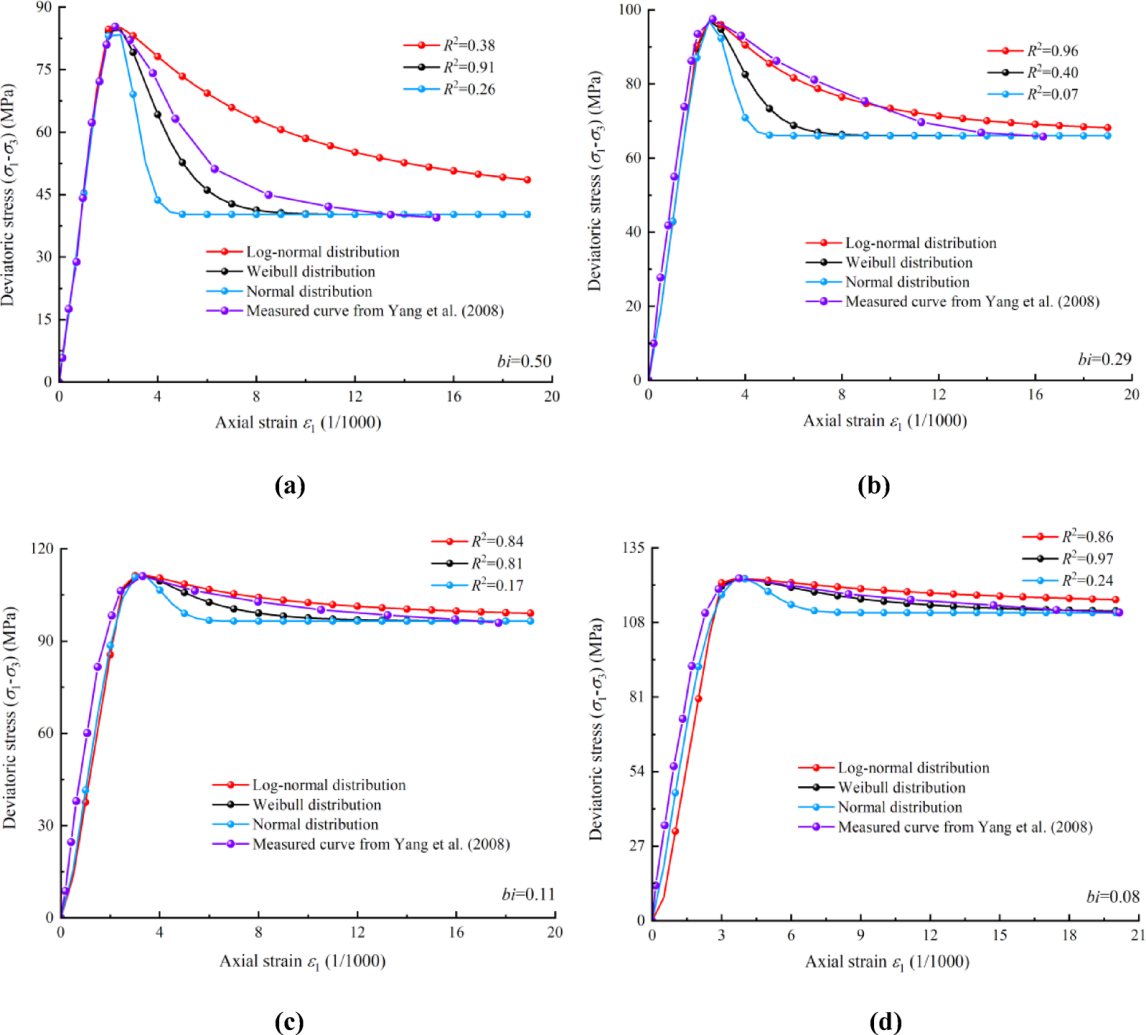
Comparison between measured curves of marble^33^ and probability distribution model curves under different confining pressures. (**a**) σ_3_ = 5.0MPa, (**b**) σ_3_ = 10.0MPa, (**c**) σ_3_ = 20.0MPa, (**d**) σ_3_ = 30.0MPa.

**Fig. 5:**
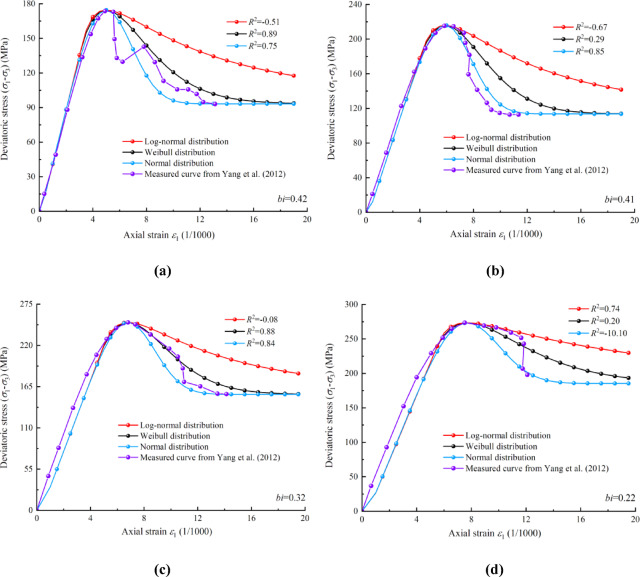
Comparison between measured curves of sandstone^34^ and probability distribution model curves under different confining pressures. (**a**) σ_3_ = 20.0MPa, (**b**) σ_3_ = 35.0MPa, (**c**) σ_3_ = 50.0MPa, (**d**) σ_3_ = 65.0MPa.

The M-C criterion was adopted to characterize the strength of micro-elements, with their strength assumed to follow three distinct probability distribution modes. Theoretical stress–strain curves of rocks under different confining pressures were obtained and subsequently compared with the measured curves in Figs. 1, 2, 3, 4, 5, where *b*_*i*_ denotes the relative brittleness index. The results reveal significant differences in the simulation of post-peak deformation behavior among theoretical models employing different probability distributions. Specifically, the magnitude of the stress drop decreases in the order: the Nd, Wd and Ld.

Similarly, when the D-P and H-B criteria were employed to characterize micro-element strength under the same three probability distribution modes, the resulting theoretical curves are omitted here for brevity. The findings indicate that the post-peak simulation performance of model curves derived from different rock strength criteria is consistent with that observed for the M-C criterion. In other words, the choice of strength criterion does not alter the post-peak stress drop patterns governed by the probability distribution modes of micro-element strength.

**Fig. 6:**
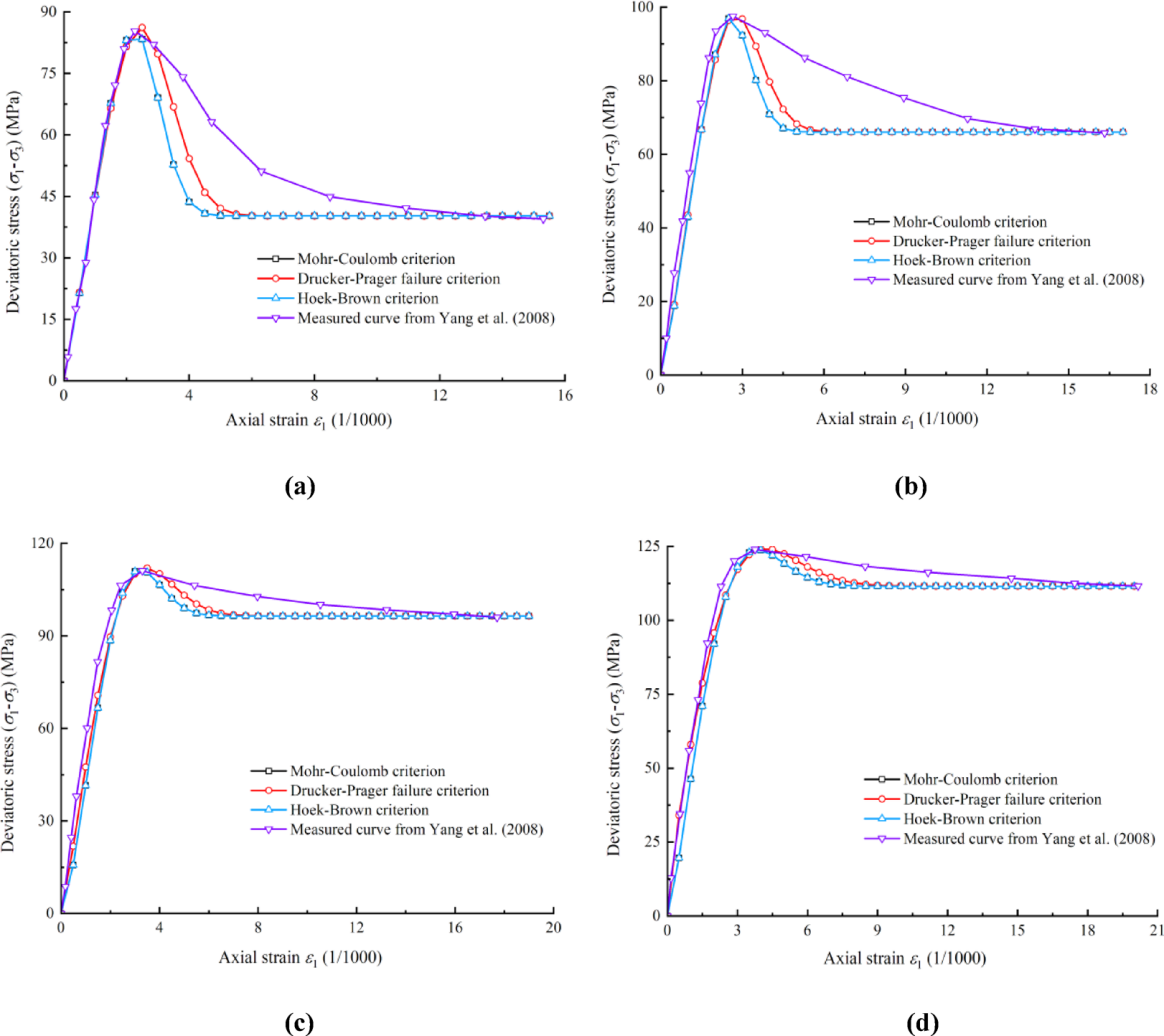
Comparison between measured curves of marble^33^ and strength criteria model curves under different confining pressures. (**a**) σ_3_ = 5.0MPa, (**b**) σ_3_ = 10.0MPa, (**c**) σ_3_ = 20.0MPa, (**d**) σ_3_ = 30.0MPa.

Further analysis was conducted to examine the influence of different strength criteria on the post-peak simulation accuracy of theoretical models under identical probability distribution modes. Due to space limitation, the simulation focused exclusively on the test curves of marble^33^, with micro-element strength assumed to follow the Normal distribution while applying the three selected strength criteria. The resulting model curves under distinct strength criteria are presented in Fig. 6. Theoretical curves based on the M-C and H-B criteria are nearly identical, whereas the model curve derived from the D-P criterion exhibits better agreement with the experimental data in the post-peak phase. Similarly, when micro-element strength was assumed to follow the Weibull and Log-normal distributions under the same three strength criteria, the corresponding curves are omitted here for brevity. The results indicate that the post-peak simulation performance of theoretical curves under different probability distribution modes follows the same pattern as that observed for the normal distribution. In other words, variations in probability distribution modes do not alter the post-peak stress drop patterns governed by the choice of strength criteria.

### Selection of probability distribution mode of micro-element strength

Different types of rocks exhibit distinct deformation characteristics, and the fundamental deformation behavior of a given type of rock is closely related to the confining pressure. As the confining pressure increases, the characteristic stress values progressively rise, while the rate of post-peak brittle stress drop gradually diminishes. In this study, brittleness is quantified using the relative brittleness index *b*_*i*_, defined as the ratio of the difference between peak stress and residual stress to the peak stress^35–37^. Table 2 presents the *b*_*i*_ for five types of rocks under different confining pressures. The results indicate that higher confining pressures consistently reduce the *b*_*i*_ of each type of rock. Increasing confining pressure promotes brittle-to-ductile transition in the mechanical behavior of rocks.

**Table 2:** Relative brittleness index *b*_*i*_ of rocks under different ***σ***_3_.

**Coal**^31^		**Norite**^31^		**Quartzite**^32^		**Marble**^33^		**Sandstone**^34^	
***σ***_**3**_**/MPa**	***b***_***i***_	***σ***_**3**_**/MPa**	***b***_***i***_	***σ***_**3**_**/MPa**	***b***_***i***_	***σ***_**3**_**/MPa**	***b***_***i***_	***σ***_**3**_**/MPa**	***b***_***i***_
3.0	0.85	3.0	0.77	3.45	0.75	5.0	0.50	20.0	0.42
5.0	0.74	5.0	0.75	6.90	0.64	10.0	0.29	35.0	0.41
8.0	0.69	8.0	0.67	13.80	0.51	20.0	0.11	50.0	0.32
–	–	–	–	–	–0	30.0	0.08	65.0	0.22

The results indicate that lower confining pressures increase brittleness, leading to notable discrepancies between the post-peak descending trends of theoretical curves derived from the three probability distribution modes and those of the test curves. Conversely, as brittleness decreases, the post-peak descending trends of the theoretical curves progressively converge with the test data. The observed differences in descending behavior among the distribution modes provide a basis for assessing the suitability of each mode in simulating test curves. To quantitatively evaluate the simulation accuracy of the post-peak theoretical curves, the coefficient of determination *R*^*2*^ is employed as the evaluation metric^38^:

47 $$R^{2} = 1 - \frac{{\sum\limits_{i = 1}^{N} {(\sigma_{1}^{{{\mathrm{theory}}}} - \sigma_{1}^{{{\mathrm{test}}}} )_{i}^{2} } }}{{\sum\limits_{i = 1}^{N} {(\sigma_{1}^{{{\mathrm{test}}}} - E[\sigma_{1}^{{{\mathrm{test}}}} ])_{i}^{2} } }}$$

in which $\sigma_{1}^{{{\mathrm{theory}}}}$ and $\sigma_{1}^{{{\mathrm{test}}}}$ represent the post-peak theoretical and test values, respectively; *N* denotes the number of test data points; $E[\sigma_{1}^{{{\mathrm{test}}}} ]$ signifies the mean of test values. Table 3 lists the probability distribution modes employed by the proposed model to obtain optimal simulation results, presented as a function of the *b*_*i*_.

**Table 3:** Optimal probability distribution mode for rocks with different *b*_*i*_*.*

*b*_*i*_	0.08	0.11	0.22	0.29	0.32	0.41	0.42	0.50
Optimal mode	Wd	Ld	Ld	Ld	Wd	Nd	Wd	Wd
*R*^*2*^	0.97	0.84	0.74	0.96	0.88	0.85	0.89	0.91

Further analysis reveals that when 0.3 < *b*_*i*_ ≤ 0.5, the Wd exhibits superior capability in simulating the post-peak stress–strain curves of rocks compared to the other two probabilistic models. This observation notably indicates that the strength distribution characteristics of the micro-elements within this range align more closely with the statistical properties of the Wd, where failure is governed by the most severely flawed micro-elements. For specimens with *b*_*i*_ = 0.41, test curve analysis indicates that significant fractures develop during the accelerated damage phase. Consequently, a sharp decline in load-bearing capacity occurs over a small strain interval, a trend that is more accurately captured by the Nd. This implies that the micro-element strength distribution aligns more closely with the statistical features of Nd under such conditions.

When *b*_*i*_ ≤ 0.3, the Ld yields optimal simulation performance, suggesting that the strength distribution of micro-elements within this range better conform to the statistical features of Ld. Notably, for specimens with *b*_*i*_ = 0.08, although the Wd provides the best fit, the theoretical curve following the Ld still achieves *R*^*2*^ = 0.86, further demonstrating its acceptable conformity to micro-element strength distribution characteristics.

When *b*_*i*_ > 0.5, the pronounced brittleness of rocks limits the applicability of three probabilistic distribution models. Even the statistical properties of the Nd where micro-defects are more uniformly distributed fail to accurately replicate the sharp post-peak stress degradation observed experimentally under such conditions. Coupling continuous statistical damage models with discontinuous approaches may provide a pathway to better simulate the rapid stress degradation characteristic of highly brittle rocks. Therefore, to improve the simulation of post-peak behavior in triaxial compression tests, the probability distribution model for micro-element strength should be selected according to the range of *b*_*i*_, as recommended in Table 4, thus achieving more reasonable post-peak simulation results. It is worth noting that the proposed selection method is derived from the analysis of five rock types under triaxial compression conditions, and its applicability to other rock types or loading conditions should be verified with additional test data. The guidelines are intended to provide a physically informed starting point for model selection rather than a universally applicable rule.

**Table 4:** Selection of optimal probability distribution mode.

*b*_*i*_	Optimal mode
*b*_*i*_ ≤ 0.3	Log-normal distribution
0.3 < *b*_*i*_ ≤ 0.5	Weibull distribution
*b*_*i*_ > 0.5	Normal distribution

## Sensitivity analysis of probability distribution parameters

As indicated by the deterministic equations governing the probabilistic distribution parameters, the two distribution parameters are uniquely determined for given confining pressure. This implies that under fixed confining pressure conditions, the model yields single theoretical curve. Consequently, theoretical curves derived from parameter calculations based on the extremum method inherently struggle to capture the stochastic variability observed in test curves. Given that the post-peak characteristics of theoretical curves are fundamentally governed by the parameters of the probability distribution function, this study conducts parametric analyses to evaluate the influence of variations in these distribution parameters on the stress degradation rate in post-peak theoretical curves under different strength criteria.

**Fig. 7:**
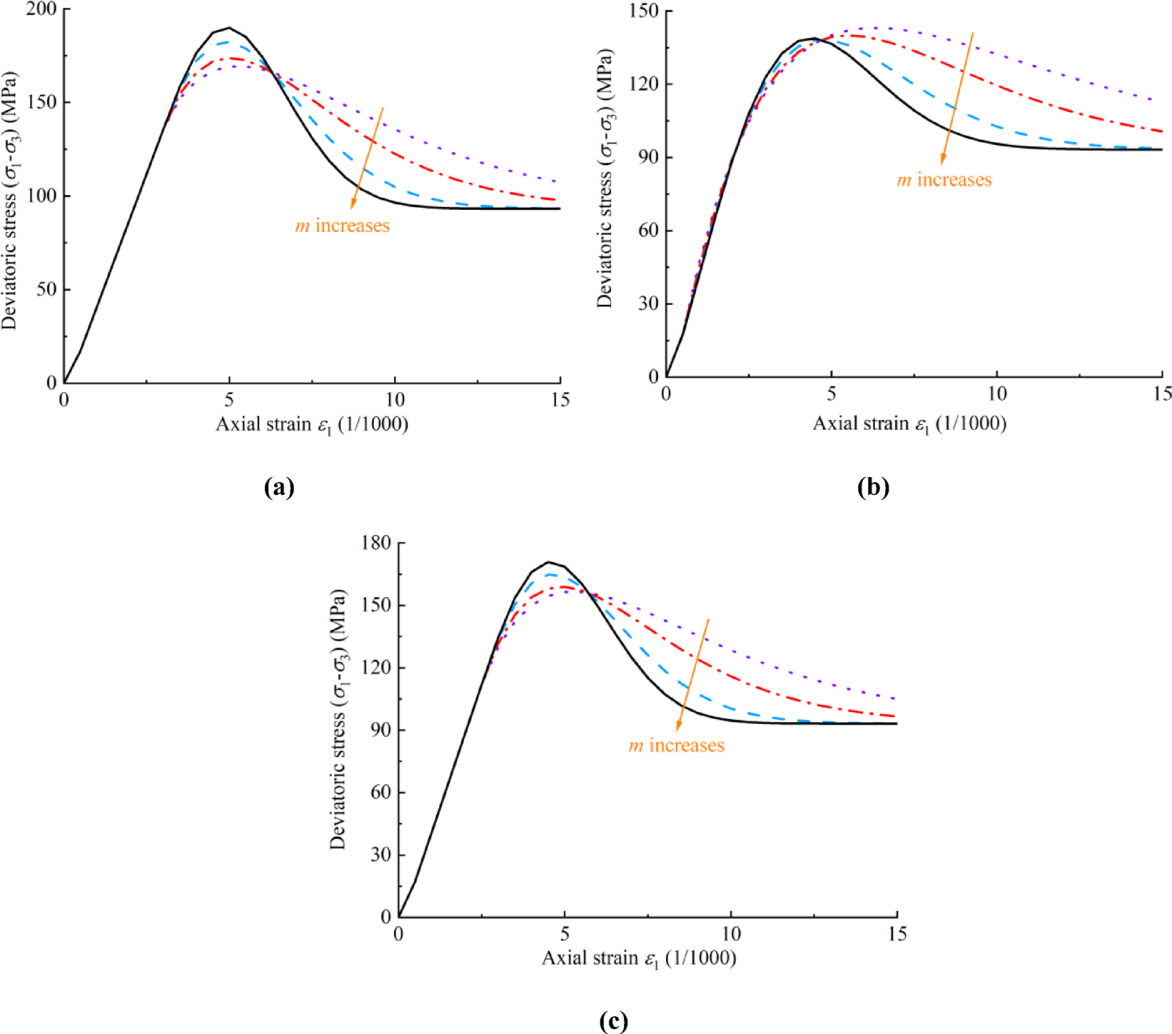
Influence of the parameter *m* on model curves under different strength criteria. (**a**) M-C, (**b**) D-P, (**c**) H-B.

**Fig. 8:**
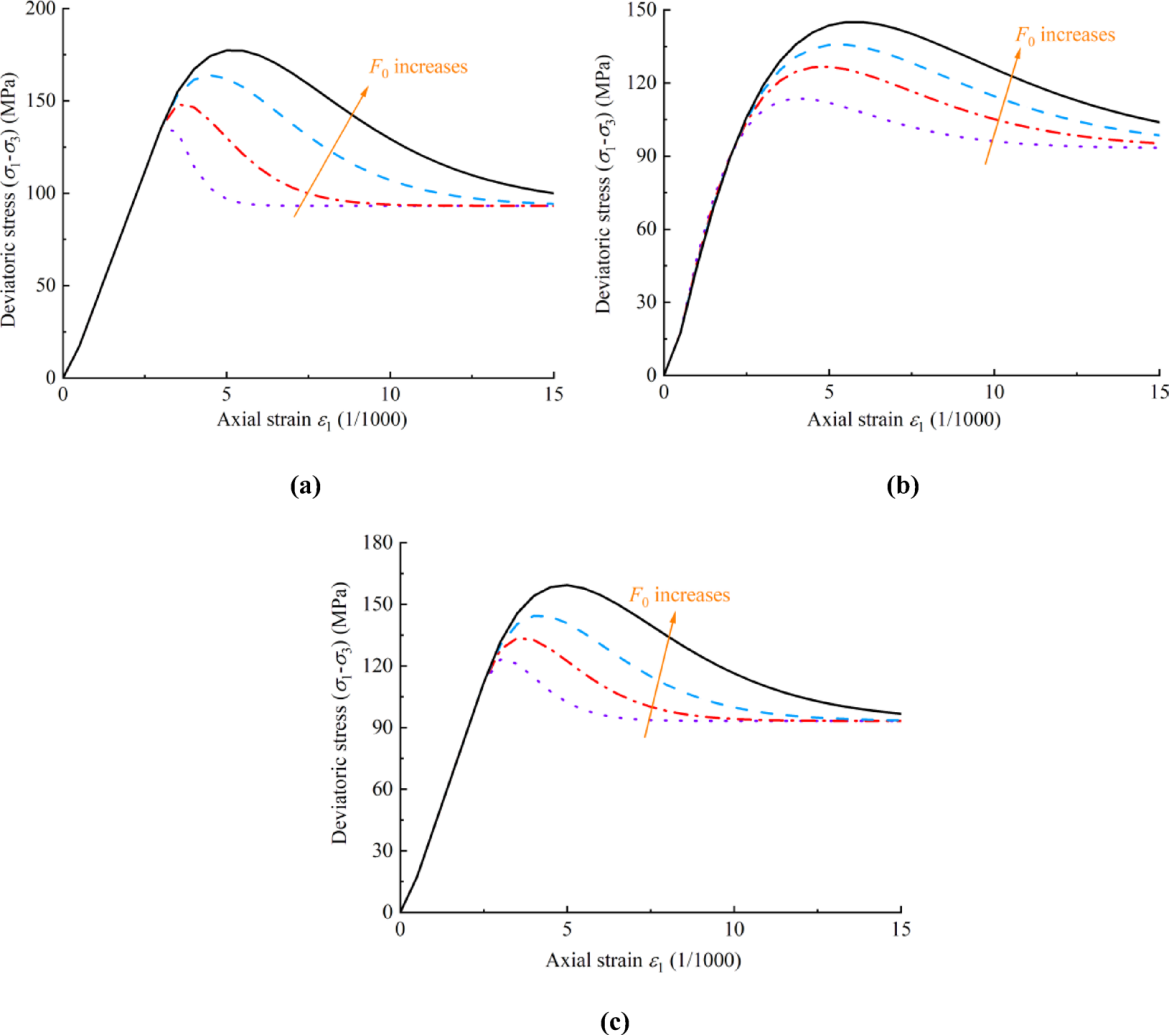
Influence of the parameter *F*_0_ on model curves under different strength criteria. (**a**) M-C, (**b**) D-P, (**c**) H-B.

As illustrated in Figs. 7 and 8 for the Wd, the parameter *m* exhibits consistent sensitivity to the strain-softening process across three strength criteria. With increasing *m*, the theoretical curves display an accelerated rate of post-peak stress degradation. In contrast, the influence of the parameter *F*_0_ on post-peak behavior varies depending on the strength criterion employed. Specifically, for the M-C criterion, larger values of *F*_0_ lead to a monotonically decreasing rate of stress degradation. For the D-P criterion, higher values of *F*_0_ result in progressively accelerating degradation. For the H-B criterion, the post-peak stress degradation rate shows negligible sensitivity to variations in *F*_0_.

**Fig. 9:**
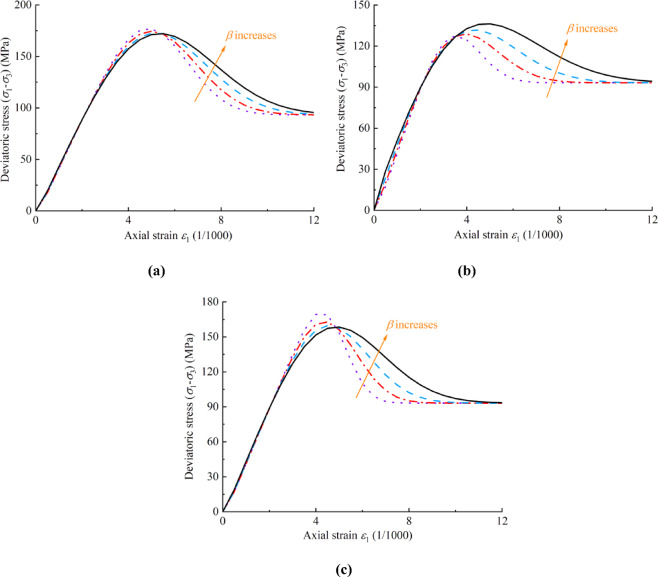
Influence of the parameter β on model curves under different strength criteria. (**a**) M-C, (**b**) D-P, (**c**) H-B.

**Fig. 10:**
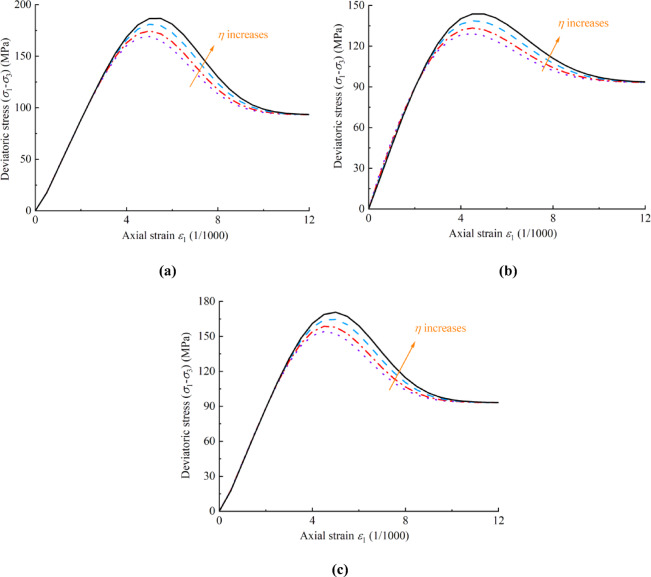
Influence of the parameter *η *on model curves under different strength criteria. (**a**) M-C, (**b**) D-P, (**c**) H-B.

Figures 9 and 10 demonstrate that for the Nd, theoretical curves exhibit consistent behavioral patterns across three strength criteria. Increasing the parameter β reduces the post-peak stress degradation rate, resulting in more gradual strain-softening characteristics. Conversely, higher values of *η* accelerate the stress degradation rate, enhancing brittle fracture. It indicates that β suppresses stress degradation by reflecting the restraining effect of micro-element strength dispersion, while *η* governs the magnitude of post-peak strength deterioration.

**Fig. 11:**
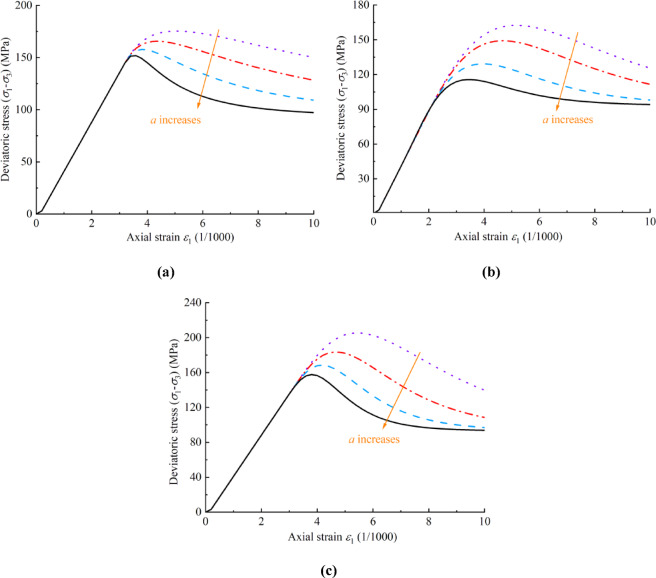
Influence of the parameter *a* on model curves under different strength criteria. (**a**) M-C, (**b**) D-P, (**c**) H-B.

**Fig. 12:**
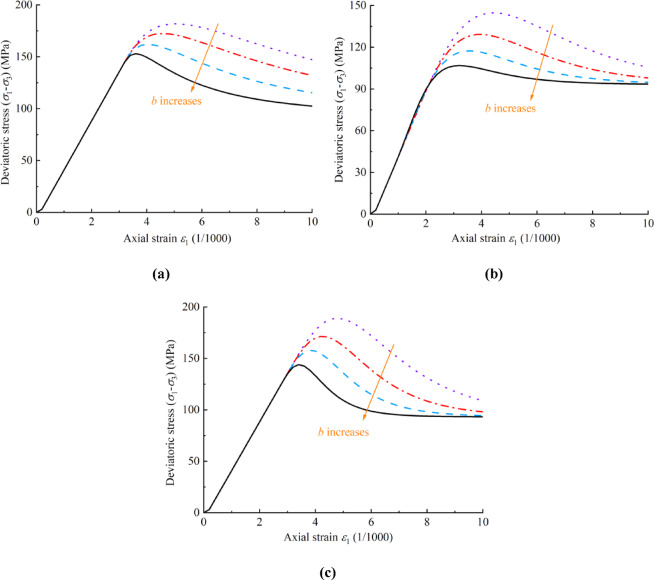
Influence of the parameter *b* on model curves under different strength criteria. (**a**) M-C, (**b**) D-P, (**c**) H-B.

Figures 11 and 12 reveal that for the Ld, increasing the parameters *a* and *b* accelerates the post-peak stress degradation rate for both the M-C and H-B criteria. In contrast, the D-P criterion exhibits a diametrically opposed trend: higher values of *a* and *b* lead to a progressively decreasing stress degradation rate. This divergence indicates that the interaction between the probabilistic distribution parameters and post-failure mechanical behavior is inherently criterion-dependent.

Consequently, only under the Nd do the probabilistic parameters exert a consistent influence on the post-peak stress degradation evolution across the three strength criteria, regardless of the strength criterion adopted. For the Wd and Ld, however, the effect of identical parameter variations is strongly criterion-dependent. Critically, the results indicate that the stochastic distribution not only governs the statistical characteristics of model parameters but can also modify the post-peak strain-softening behavior through its interaction with the strength criterion. The parametric consistency observed under the Nd reflects its capacity to capture macro-scale fracture homogeneity when micro-defects are uniformly distributed. Conversely, the contrasting responses under the Wd and Ld reveal a heightened sensitivity to the selected strength criterion when the micro-element strength distribution is asymmetric. These findings underscore the critical importance of appropriately selecting measurement method of micro-element strength and integrating experimental statistical data to determine the optimal distribution in rock engineering analyses, thereby improving simulation accuracy for fracture behavior of brittle rocks.

## Dual-parameter collaborative correction method

The post-peak evolution of theoretical curves is fundamentally governed by the parameters of the probabilistic distribution. Under a given confining pressure, these parameters are uniquely determined, which inherently constrains the coefficient of determination *R*^2^ from consistently attaining high level. To rapidly enhance predictive accuracy of theoretical models for post-peak curves, this study proposes a dual-parameter collaborative optimization approach.

The findings presented above demonstrate that identical parameter variations exert criterion-dependent influences on post-peak behavior for the Wd and Ld. Directly incorporating strength criteria into the optimization process would not only increase methodological complexity but also reduce the generalizability of the approach. Therefore, rather than differentiating among strength criteria, the proposed method prioritizes parametric interdependence. By collaboratively adjusting the distribution parameters, the theoretical curves can be better aligned with experimental data, thereby elevating the coefficient of determination *R*^2^.

**Fig. 13:**
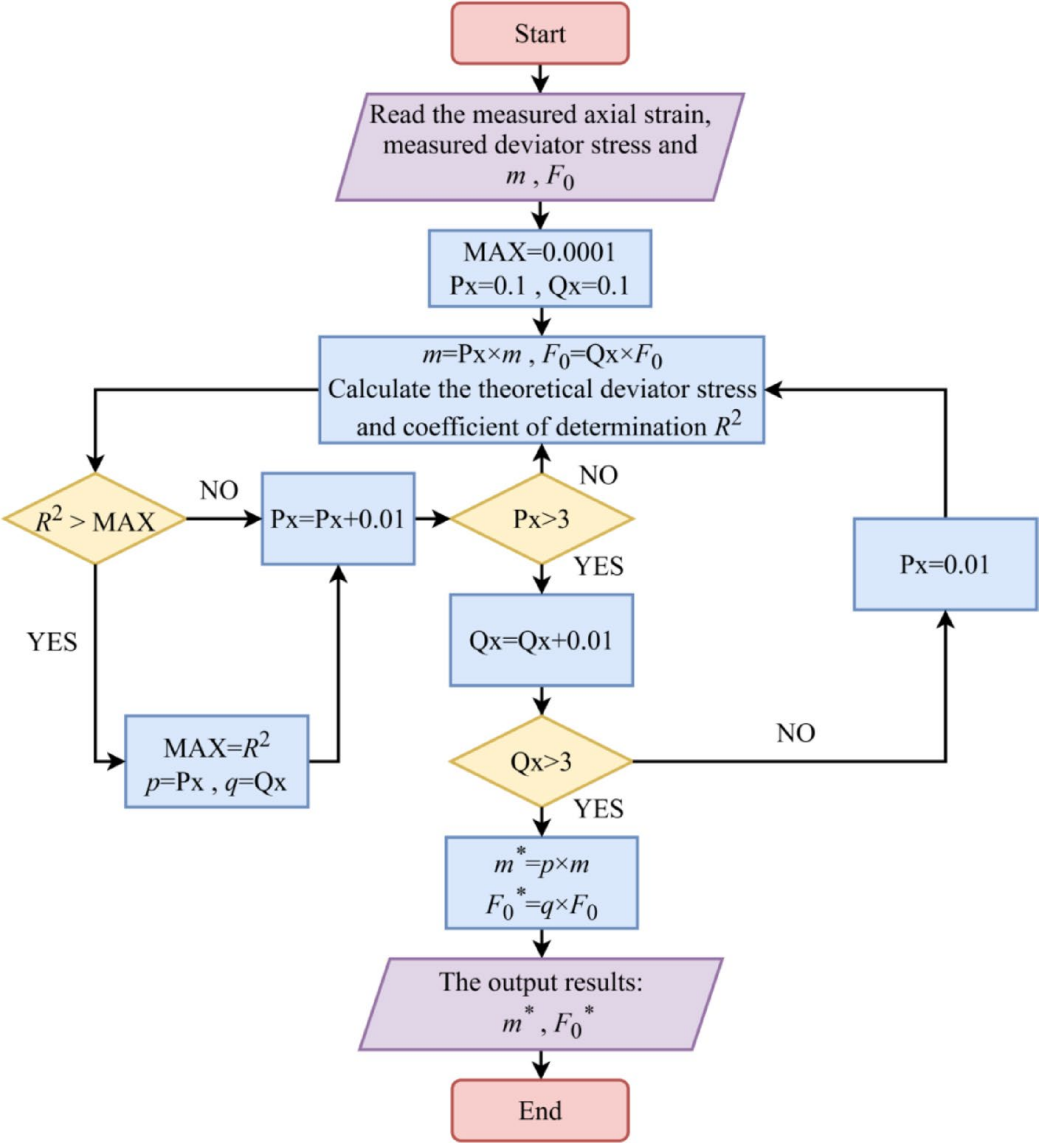
Process flow for collaborative optimization of the parameters *m* and *F*_0_.

Specifically, adopting the M-C criterion and assuming the Wd, this study implements collaborative optimization of the parameters *m* and *F*_0_. The optimized parameters $m^{ * }$ and $F_{0}^{ * }$ are derived through adjustment ratios *p* and *q* for *m* and *F*_0_, respectively, as diagrammed in Fig. 13. For model parameters derived from other combinations of strength criteria and probability distributions, the collaborative optimization procedure follows an analogous approach and is therefore omitted here for brevity. It should be emphasized that the dual-parameter collaborative correction method is proposed as an engineering-oriented calibration approach rather than a mechanism-based enhancement.

**Fig. 14:**
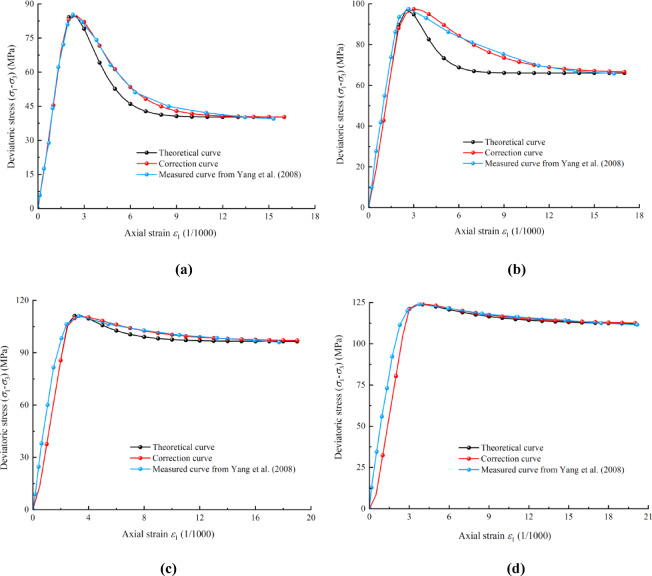
Comparison between measured and optimized theoretical curves of marble^33^ under different confining pressures. (**a**) σ_3_ = 5.0MPa, (**b**) σ_3_ = 10.0MPa, (**c**) σ_3_ = 20.0MPa, (**d**) σ_3_ = 30.0MPa.

**Fig. 15:**
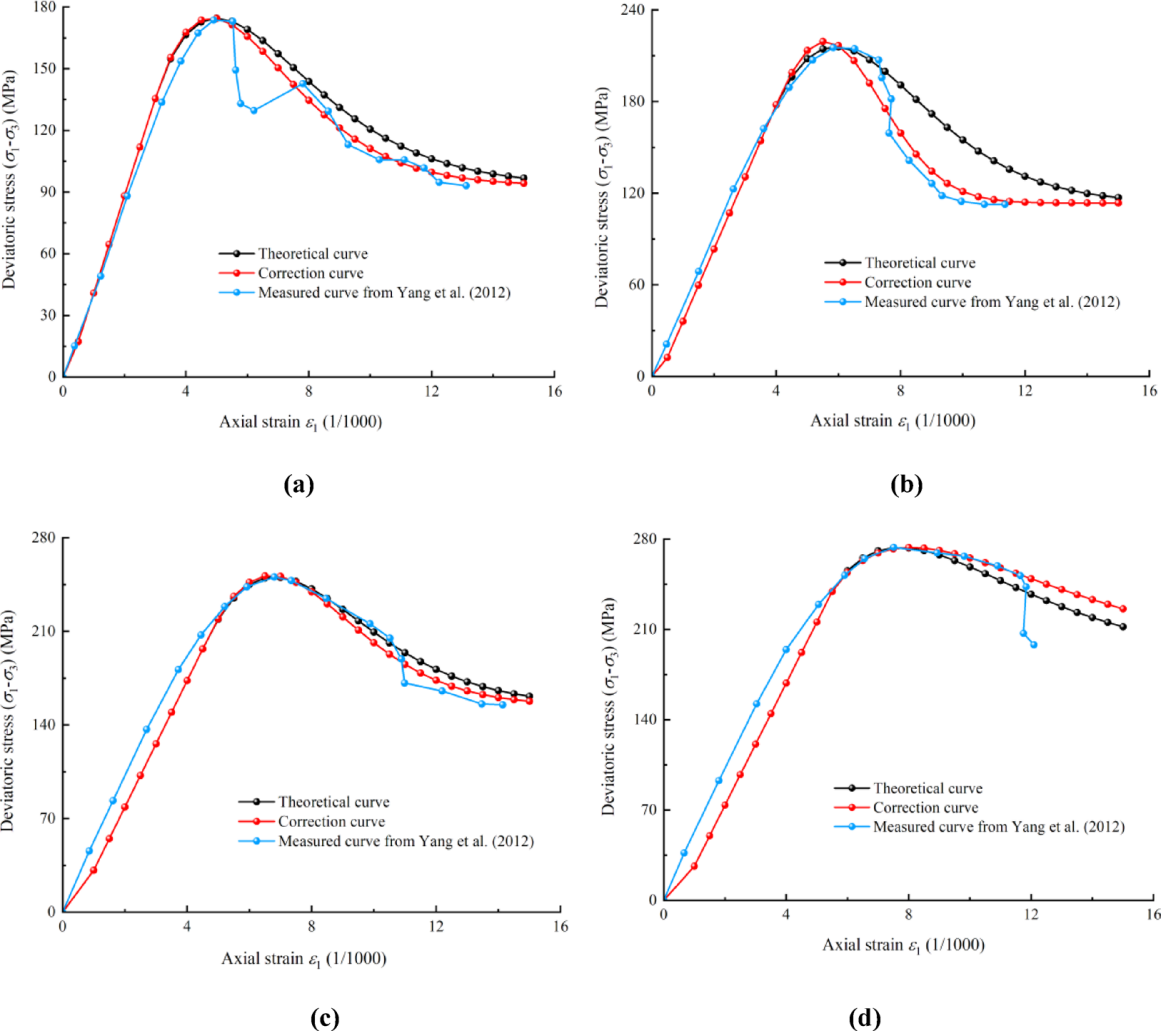
Comparison between measured and optimized theoretical curves of sandstone^34^ under different confining pressures. (**a**) σ_3_ = 20.0MPa, (**b**) σ_3_ = 35.0MPa, (**c**) σ_3_ = 50.0MPa, (**d**) σ_3_ = 65.0MPa.

To validate the effectiveness of the proposed optimization method, a comparative analysis was conducted between the original and optimized theoretical curves for marble and sandstone, as shown in Figs. 14 and 15. The results demonstrate that the dual-parameter optimization significantly improves the agreement with test curves during the post-peak phase, thereby confirming its superior efficacy in enhancing the simulation accuracy of the proposed model. It should be noted that the proposed method is primarily intended for simulating post-peak behavior based on experimentally calibrated parameters, rather than purely serving as a predictive model.

## Conclusions

To accurately simulate the complete fracture-to-failure process of brittle rocks, this study quantitatively investigated the evolution of stress–strain curves under multiple strength criteria and probability distributions, and specifically developed a dual-parameter collaborative optimization method. The main conclusions are summarized as follows:

1. The simulation accuracy of post-peak stress–strain curves is critically dependent on the selection of the probability distribution, whereas the choice of strength criterion exerts only a minor influence on overall predictive performance. Specifically, the Wd achieves optimal performance for 0.3 < *b*_*i*_ ≤ 0.5, the Ld for *b*_*i*_ ≤ 0.3 and the Nd for *b*_*i*_ > 0.5, but it remains qualitatively inadequate in capturing post-peak descent rate.
2. Parametric impacts on theoretical curves exhibit significant correlation with distribution patterns. For the Wd and Ld, parametric variations induce distinctly different post-peak behaviors across strength criteria. In contrast, parametric variations exhibit negligible influence on post-peak simulation efficacy regardless of strength criterion alternations for Nd.
3. The proposed dual-parameter collaborative optimization method enhances simulation accuracy across distribution modes. It eliminates interference from strength criterion during parameter calibration and maintains its applicability across multiple probability distribution modes.

## Data Availability

The data used and/or analyzed during the current study available from the corresponding author on reasonable request.
